# Using a Heuristic Tool to Improve Symptom Self-Management in Adolescents and Young Adults With Cancer: Protocol for a Randomized Controlled Trial

**DOI:** 10.2196/76667

**Published:** 2025-12-24

**Authors:** Suzanne Ameringer, Grace Hodges, R K Elswick Jr, Lauri Linder, Catherine Fiona Macpherson, Kristin Stegenga

**Affiliations:** 1 Department of Adult Health and Nursing Systems, School of Nursing, Virginia Commonwealth University, 1100 East Leigh Street, Richmond, VA, 23298, United States, 1 804-828-5151; 2 Health Systems and Science Across the Lifespan, College of Nursing, University of Utah, Salt Lake City, UT, United States; 3 Pediatrics, Seattle Children's Hospital, Seattle, WA, United States; 4 Pediatrics, Children's Mercy Hospital, Kansas City, MO, United States

**Keywords:** randomized clinical trial, cancer, adolescents and young adults, digital health, symptom management, mobile phone

## Abstract

**Background:**

Adolescents and young adults (AYAs) with cancer experience multiple distressing symptoms during treatment, yet few developmentally relevant resources have been developed to help them self-manage their symptoms. Empowering patients to have a more active role in self-management during cancer treatment may lessen their symptom severity and distress.

**Objective:**

The aim of this study is to test an intervention designed to improve symptom self-management, the Computerized Symptom Capture Tool (C-SCAT), by helping AYAs understand their unique symptom experience and discuss it with their health care providers.

**Methods:**

We are conducting a multisite, 2-group randomized controlled trial to evaluate the effects of the C-SCAT in improving symptom self-management versus usual care in 126 AYAs who are within the first 3 months of a cancer diagnosis and who are receiving chemotherapy. Participants are randomly assigned to either the C-SCAT intervention group or the usual care group. The primary aim is to determine the effects of the C-SCAT versus usual care on the primary outcomes of self-efficacy for symptom management and symptom self-management behaviors. The secondary aim is to examine the effects of the C-SCAT versus usual care on distal outcomes, including symptoms and quality of life. Participants complete measures of self-efficacy for symptom management, symptom self-management behaviors, symptom severity and distress, quality of life social function, and quality of life satisfaction of social function at baseline (time 0), immediately postintervention (time 1), and at follow-up 1 month later (time 2).

**Results:**

Recruitment started on January 4, 2024, at the first site, and all sites were open by May 1, 2024. Thus far, accrual has been set at 73% of the quarterly benchmark goal. Linear mixed effects models will be used to test for group differences across time for the primary and secondary aims.

**Conclusions:**

This randomized controlled trial is evaluating an innovative, point-of-care intervention designed to be used at a clinic visit with the provider to improve symptom self-management for AYAs with cancer.

## Introduction

### Background

Approximately 90,000 adolescents and young adults (AYAs) are diagnosed with cancer yearly in the United States [1]. As a distinct subset of patients with cancer, AYAs are underserved, understudied, and at risk for inferior outcomes, both physical and psychosocial, compared to younger children and older adults [2]. The epidemiology of AYA cancers, the physical and psychosocial effects of the requisite treatments, and characteristics of the AYAs’ developmental stage combine to create a distinctive cancer experience, including multiple distressing interrelated symptoms [3-5].

Undermanaged, persistent, and recurrent symptoms negatively affect quality of life, function, health care costs, morbidity, and survival [6]. Specifically, AYAs report a decline in both mental and physical aspects of quality of life with as few as 3 symptoms [7]. Symptoms can interfere with the AYA’s ability to attend school, participate in sports, and develop and maintain romantic relationships [8]. AYAs with cancer desire personalized symptom management information [9], but communicating their symptom experience to health care providers (HCPs) can be difficult for multiple reasons. These include the AYA’s lack of familiarity with the health care environment, inexperience in talking about symptoms, a lack of systematic symptom assessment in many clinical settings, and the HCP’s focus on issues other than those important to the AYA [10]. One critical aspect of obtaining relief from symptoms is to enhance an individual’s ability to self-manage symptoms.

Self-management is defined as the process by which individuals use knowledge, beliefs, skills, and abilities to achieve health-related outcomes [11]. A distinct component of self-management is symptom self-management, defined as “a dynamic, self-directed process of implementing behaviors that recognize, prevent, relieve, or decrease the timing, intensity, distress, concurrence, and unpleasant quality of symptoms to achieve optimal performance outcomes ” [12]. Developing self-management abilities in general and symptom self-management abilities specifically may be challenging for AYAs with cancer for several reasons. They may be gaining autonomy but often will still be somewhat dependent on parents or guardians. They may be emotionally or physically unprepared for or incapable of executing the responsibilities they need to assume. Finally, they are basically novices at dealing with chronic illness, monitoring symptoms, and interacting with HCPs [12]. While effective interventions exist for AYAs to improve self-management of other chronic diseases, such as asthma and diabetes [13], nearly none exist for AYAs with cancer. Two aspects that are critical to improving symptom self-management are having self-efficacy to manage symptoms and engaging in effective symptom self-management behaviors.

The concept of self-efficacy is described as the confidence a person has in their ability to engage successfully in a behavior [14] and is essential to self-management [15]. Self-efficacy for symptom self-management is defined as the individual’s belief in their “ability to implement behaviors to prevent, recognize, and relieve symptoms” [16]. In AYAs with cancer, evidence suggests that increased self-efficacy is associated with improved symptom self-management behaviors, in particular improved nutrition and physical activity [17,18]. Among adults with cancer, self-efficacy is a key predictor of symptom self-management behaviors and is associated with decreased symptom severity or distress, improved psychosocial function, and greater quality of life [19-23]. Targeting self-efficacy has been shown to be effective; however, interventions to increase AYAs’ self-efficacy for symptom self-management are lacking.

Symptom self-management behaviors are those actions that individuals initiate to alleviate symptoms [24]. Mastering effective symptom self-management behaviors can be particularly challenging as AYAs transition from having their parents manage their health care to managing it on their own [25]. Studies addressing symptom self-management behaviors among AYAs receiving cancer treatment are largely cross-sectional and descriptive; however, they provide important insights into the strategies AYAs use. AYAs may initiate symptom self-management behaviors to target an individual symptom, such as taking an antiemetic medication to manage nausea or to target more than 1 symptom, such as allowing for rest periods to self-manage fatigue and pain [5,26]. AYAs have revealed their rationales for engaging in certain symptom self-management behaviors, which have included enduring a given symptom for the sake of avoiding another. For example, AYAs may opt to omit opioid analgesics to avoid side effects such as nausea and vomiting [5,27]. Symptom self-management behaviors may also be influenced by the AYA’s own role-related responsibilities; for example, AYAs who are parenting may select or even forgo given symptom self-management strategies in deference to priorities for their children [28].

Collectively, these studies indicate the efforts that AYAs initiate to self-manage their symptoms. More significantly, they reveal challenges that AYAs face when deciding how to optimally manage their symptoms, and therefore, a person-centered approach to support symptom self-management is vital.

Interventions to improve symptom self-management for AYAs are sparse. Limitations in research to date include an emphasis on single symptoms [29], single-site studies with small samples that often include children along with AYAs [29], and early phase testing of interventions [29,30]. Furthermore, most of the interventions are not grounded in self-management theory that aims to target critical processes such as self-efficacy, self-regulation, and communication. Two technology-based interventions to improve cancer symptom self-management were found to be feasible and acceptable to AYAs, but rigorous testing of effectiveness has not been reported [29,30]. One is a mobile phone–based symptom management system for AYAs that was piloted to incorporate feedback from AYAs into the system [30]. The other is a smartphone app for self-managing cancer-related pain that showed improved pain outcomes [29,31,32]. In summary, the lack of randomized controlled trials (RCTs) that test the effectiveness of technology-based interventions to improve symptom self-management that are tailored to AYAs with cancer is a major weakness of previous research.

To address this need, we developed and pilot tested the Computerized Symptom Capture Tool (C-SCAT), an innovative, inductive, heuristics-based symptom self-management intervention delivered via a tablet computer [33]. Heuristics are conscious or unconscious “mental models” used in decision-making and problem-solving. A heuristic approach is proposed to assist individuals in becoming more aware of their symptoms and the need to engage in symptom self-management behaviors.

The C-SCAT is a unique and efficient intervention because it is designed to be completed immediately before an HCP encounter to help AYAs better understand their symptom experience and plan how to discuss their symptoms with an HCP, including their priority symptoms and symptoms they may feel embarrassed about. In addition, the use of heuristics contrasts with typical checklist approaches to symptom assessment, which do not incorporate the patient’s perspective to identify symptoms of high priority, defined as the symptoms that are most important to them, and to identify relationships among symptoms. Use of the C-SCAT prepares AYAs to have a more focused discussion about their symptoms and self-management strategies with their HCP. The C-SCAT is unique in leveraging digital technology in a novel, developmentally relevant way to advance a person-centered health care approach to symptom self-management [34].

### Theoretical Framework

**Figure 1.**
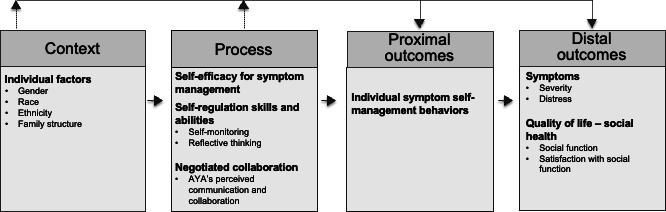
Conceptual framework. AYA: adolescent and young adult.

The Individual and Family Self-Management Theory (IFSMT) [11], which acknowledges the complex process of changing health behaviors to self-manage symptoms related to an illness, such as cancer, was used to develop the C-SCAT intervention. The theory proposes that context and process factors influence proximal and distal health outcomes. Individual contextual factors may add risk or provide protection for engagement in self-management, and process factors facilitate change in health behaviors. Interventions to improve symptom self-management can target context and process factors. This study uses a modified version of the IFSMT (Figure 1).

The C-SCAT targets three key process factors: (1) self-efficacy for symptom management, (2) self-regulation, and (3) negotiated collaboration. Self-efficacy for symptom management is enhanced by using the C-SCAT because creating the image facilitates the individual’s interpretation of their symptoms. When individuals understand their symptoms’ relationships and causes, they can engage more confidently in appropriate self-management behaviors [35]. The C-SCAT promotes self-regulation by facilitating individuals’ understanding of their symptoms so that they can create a mental model that includes (1) which symptoms are deemed ‘priority’ and need priority management, (2) how their symptoms “cluster” together, and (3) how they might approach management options. These actions facilitate processes of self-regulation, including self-monitoring, reflective thinking, and self-evaluation [11,36]. Negotiated collaboration is facilitated by the C-SCAT because its use prepares AYAs for their discussion with an HCP and provides a strategy to efficiently communicate their priority symptom issues [37,38]. An advantage of the C-SCAT is that it is designed for AYAs to share information with any HCP who is involved with their symptom management (eg, physicians, advanced practice nurses, and staff nurses). The context factors include gender, race, ethnicity, and family structure. The proximal outcome, symptom self-management behaviors, is those activities used to prevent, monitor, manage, and communicate symptoms [39]. The distal outcomes are symptom severity and distress and quality of life–social health.

For this study, the primary outcomes are self-efficacy for symptom self-management and engagement in symptom self-management behaviors. While self-efficacy for symptom self-management is a process factor in the IFSMT, it is critical to improving symptom self-management and therefore is one of the primary outcomes. The secondary outcomes are symptom severity, symptom distress, social function, and satisfaction with social function.

### Aims or Hypotheses

This RCT is testing a heuristics-based intervention designed to enhance symptom self-management in AYAs with cancer. Primary aim 1 is to determine the effects of the C-SCAT versus usual care on the primary outcomes of self-efficacy for symptom management and symptom self-management behaviors immediately postintervention (time 1) and at follow-up 1 month later (time 2). Aim 1 hypotheses are that AYAs using the C-SCAT will have higher self-efficacy for symptom self-management and higher scores on symptom self-management behaviors compared to AYAs receiving usual care. Primary aim 2 is to examine the effects of the C-SCAT versus usual care on secondary outcomes (eg, symptom severity, symptom distress, social function, and satisfaction with social function) immediately postintervention (time 1) and at follow-up 1 month later (time 2). Aim 2 hypotheses are that AYAs using the C-SCAT will have lower symptom distress and symptom severity and higher social function and satisfaction with social function compared to AYAs receiving usual care.

## Methods

### Ethical Considerations

This study was approved by Advarra on October 27, 2023 (Pro00070338), which serves as the single institutional review board of record and has received administrative approval from the institutional review boards at each study site. Privacy is protected with data stored in a secure, HIPAA (Health Insurance Portability and Accountability Act)-compliant server at the primary institution.

The informed consent process is conducted by study staff and includes verbally reviewing the consent form, answering questions, and obtaining signatures from the participant and 1 parent or legally authorized representative of participants who are minors. Obtaining informed consent or assent and parental permission occurs in person in a private setting in the inpatient or outpatient oncology setting. Assent is documented by the participant who is aged <18 years signing the informed consent, permission, or assent form. A reconsent process will occur for participants who are minors at enrollment but become adults during the study period and are reconsented as adults. Written informed consent or assent and parental permission, as appropriate, are obtained before completing baseline measures. After the informed consent process is complete, participants are considered enrolled and assigned a study identification number linked to their survey responses and intervention visits. Participants are compensated with a US $25 prepaid gift card after completing each of the first and second data collection visits and a US $30 prepaid gift card after completing the third data collection visit, for a total of US $80.

### Recruitment

At each of the sites, recruitment occurs in both inpatient and outpatient oncology settings. Potential participants are approached in person. Sites using Epic are also able to provide a recruitment message through the electronic medical record.

### Screening

At each site, oncology clinic schedules are reviewed at least weekly to identify potential participants. In addition, hospital staff in inpatient and outpatient oncology settings are asked to identify potentially eligible participants. A screening form is completed before enrollment to determine eligibility.

### Trial Design

**Figure 2.**
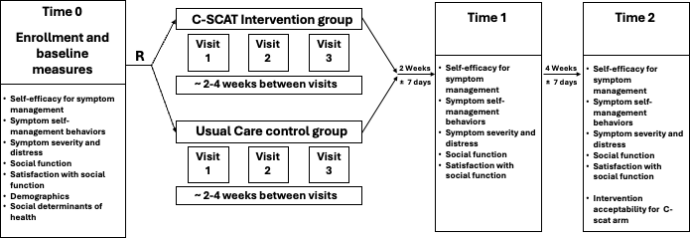
Study schema. C-SCAT: Computerized Symptom Capture Tool.

This study uses a multisite 2-group RCT design to evaluate the effects of the C-SCAT for improving symptom self-management versus usual care in AYAs who are within the first 3 months of a cancer diagnosis and who are receiving cancer treatment. For this study, cancer treatment is defined as a prescribed infusion (eg, chemotherapy or immunotherapy). Data collection occurs at 3 time points (times 0, 1, and 2). Time 0 (baseline, followed by randomization) occurs at or near the enrollment visit. The C-SCAT intervention period is approximately 12 weeks, depending on the participant’s treatment schedule. Time 1 (postintervention) occurs approximately 2 weeks (±7 d) after completion of the intervention. Time 2 (follow-up) occurs approximately 4 weeks (±7 d) after time as seen in Figure 2.

### Participants and Setting

Recruitment and enrollment are conducted by trained study staff and take place at 4 institutions from the Northwest, Intermountain West, Midwest, and Southeast regions of the United States. Participants are AYAs with cancer who are in active treatment. The inclusion criteria are as follows: (1) ages 15 to 29 years; (2) has received at least one cycle of cancer treatment and is within 3 months of receiving that first cycle of treatment; (3) receiving regularly scheduled cancer treatment and will be receiving at least three more cycles; (4) reports at least one symptom related to cancer or its treatment; and (5) is able to speak, read, and write English as required for completion of the C-SCAT and study measures. The exclusion criterion is cognitive or physical inability to complete study measures.

### Randomization

Participants are randomized to either the C-SCAT intervention or usual care control group (UCCG). Study staff and participants are not blinded to treatment allocation due to the nature of the intervention. Thus, to reduce bias, deidentified data with dummy codes for assigned conditions are used in the database to ensure blinded data analysis. Using stratified randomization (a separate randomization at each clinical site), participants are randomized to the C-SCAT group or to the UCCG. Within each clinical site, a block randomization schema is generated with randomly selected block sizes of 2, 4, and 6. Stratified and block randomization with randomly selected block sizes are preferred in unblinded studies because they make it very difficult, if not impossible, for people enrolling participants to know what group assignment is next [40]. The biostatistician generates the various randomization schemas by clinical site, and the data manager uploads them into REDCap (Research Electronic Data Capture), allowing study staff at all sites to instantaneously receive the group assignment via computer.

### Study Intervention

The C-SCAT is a symptom heuristic tool that is delivered via a tablet computer and was developed based on a symptom cluster heuristics interview guide created by Woods et al [41]. The heuristics approach is used because it promotes AYA’s self-awareness of their symptoms, specifically it helps them visualize the ‘big picture’ of their unique symptom experience and be made aware of the need to engage in symptom self-management behaviors. Thus, the completion of the C-SCAT is the intervention.

**Figure 3.**
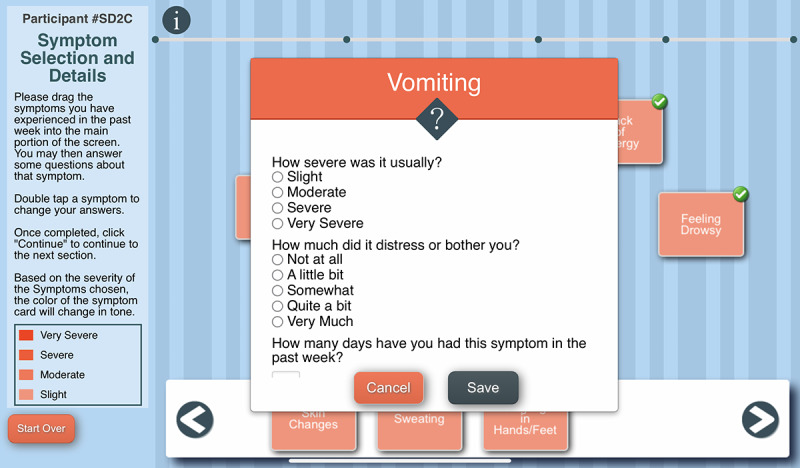
The Computerized Symptom Capture Tool selection of symptoms screen.

**Figure 4.**
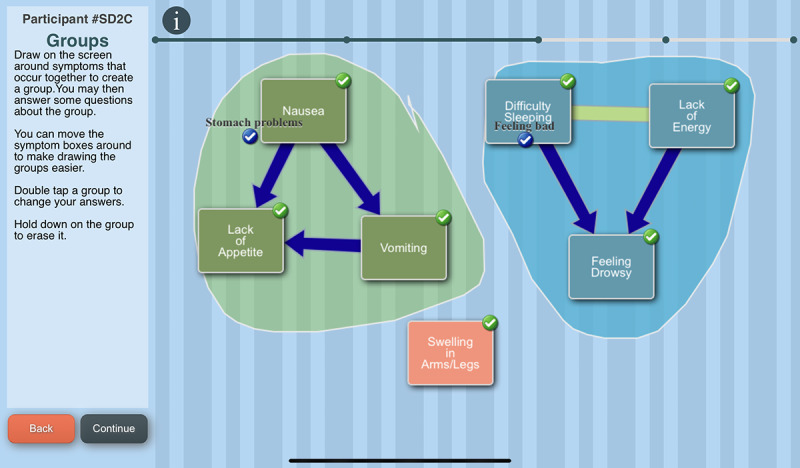
The Computerized Symptom Capture Tool identification of groups screen.

**Figure 5.**
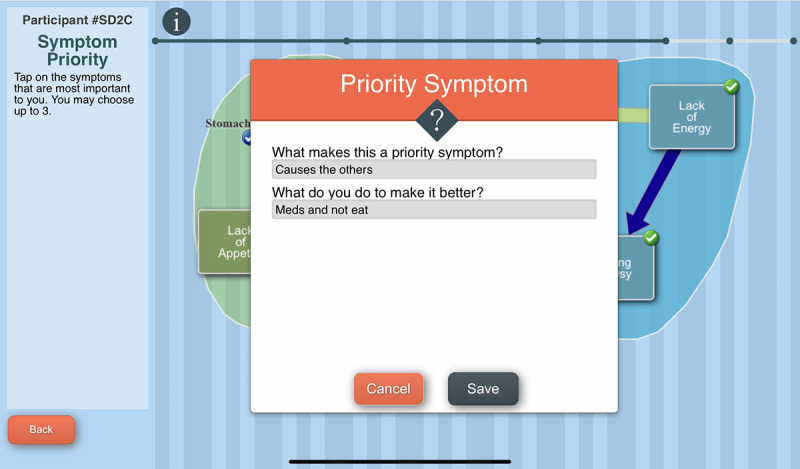
The Computerized Symptom Capture Tool identification of priority symptoms screen.

**Figure 6.**
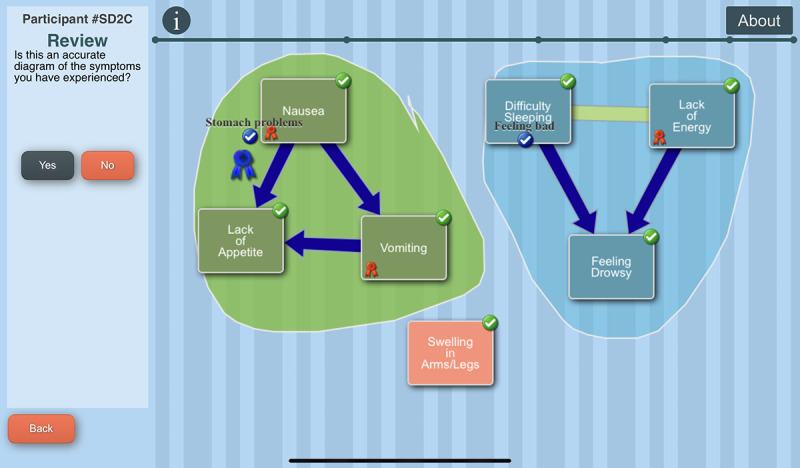
The Computerized Symptom Capture Tool review of final image screen.

The tool engages the AYA in creating a graphical image of the symptoms they experienced during the past week, designating relationships among symptoms and identifying priority symptoms. The C-SCAT includes the 32 symptoms from the Memorial Symptom Assessment Scale (MSAS) [42]. Users select symptoms they have experienced over the past week, rate each symptom’s severity and distress, and name a perceived cause (Figure 3). They then identify temporal and causal relationships between symptoms using lines and arrows, designate groups, that is, “clusters” of symptoms, and give a name to each cluster (Figure 4). Users can designate up to 3 priority symptoms (symptoms most important to them). They are asked the reason for a symptom’s designation as a priority symptom and what they do to alleviate that symptom (Figure 5). Next, they are asked to designate a priority cluster and finally to confirm whether the image accurately reflects their symptom experience (Figure 6).

The C-SCAT is completed just before an HCP visit to assist the AYA in understanding their symptom experience and communicating it effectively to the HCP to facilitate symptom self-management. Once they complete the C-SCAT, the image is printed out for them. Although they are encouraged to share the C-SCAT image with their providers, it is their decision as to whether they choose to share it.

### Description of the C-SCAT Intervention Group

Participants assigned to the C-SCAT intervention group complete the C-SCAT on a tablet computer in addition to their institution’s usual care and processes for assessing symptoms before three of their scheduled clinic visits with their HCPs for their cancer treatment. Depending on the individual’s treatment protocol, treatments are typically every 2 to 4 weeks. Before the AYA’s first encounter with the HCP, study staff provide a short interactive practice session on how to use the C-SCAT that includes (1) an introduction to the C-SCAT as a tool that can be used to create a graphical image of the AYA’s symptom experience that can be printed out and shared with the HCP, (2) a video that demonstrates how to complete the C-SCAT and how it can be used in an encounter with the HCP, (3) a practice session to ensure that the AYA is proficient and comfortable using the C-SCAT, and (4) a question and answer period. The practice session takes approximately 5 minutes. The AYA completes the C-SCAT at each of 3 HCP (intervention) visits, and the resulting image is printed out and given to the AYA to review and to share the image with the HCP as they wish. As this study is focused on improving symptom self-management and although study staff encourage the AYA to share the C-SCAT image with their HCP, ultimately, it is up to the AYA to decide whether they want to share the image. Before the start of the study, study staff gave in-services to the oncology clinics’ HCPs on how to interpret the C-SCAT image and also supplied the HCP with a printed guide as to how to interpret the C-SCAT image at each participant intervention study visit.

### Description of the UCCG

Participants assigned to the UCCG receive care based on the institution’s usual approach to assessing symptoms during the HCP encounter. To provide attention control to the UCCG and help prevent disproportional attrition from that group, study staff make contact with UCCG participants 3 times during the intervention period (same as the intervention group) during scheduled clinic visits and communicate the following: (1) express appreciation for ongoing study participation, (2) ask how everything is going with their treatment, (3) ask if anything has changed with their treatment plan since the last study visit, and (4) confirm continued willingness to participate in the study. None of the recruitment sites use any standardized approach to assess multiple cancer symptoms, although all sites routinely assess for pain. Several sites use depression screening tools, but only for symptoms related to depression. Thus, it is not anticipated that elements of usual care will overlap with the C-SCAT intervention.

### Intervention Fidelity and Training of Study Staff

Intervention fidelity is guided by recommendations from the National Institutes of Health Behavior Change Consortium [43], with attention to design, training, delivery, receipt, and enactment. Study staff at each site are involved in recruitment, retention, and data collection. Training addresses protection of human subjects, research compliance, study methods, data entry and management, roles and responsibilities, training on the C-SCAT intervention, and attention to the UCCG. All staff complete required human subjects and other relevant training at initiation of the project and throughout as appropriate.

To ensure that the intervention is delivered as intended within and across sites, we developed a standard operating procedure manual with core procedures for all sites and specific considerations within sites and ensured training for the study staff on procedures and interventions through review of the manual, didactic sessions, and demonstrations. To maintain consistency of training, study procedures based on the manual are regularly reviewed with the study staff. To mitigate bias by study staff during study visits for both arms, we have carefully scripted each study visit to support consistent delivery. The project coordinator monitors intervention delivery and receipt by routine random checking for any differences in study logs, completion of the C-SCAT, and completeness of measures. Retraining is instituted as needed. The research team holds twice monthly meetings (and ad hoc as needed) with study staff to discuss any issues with study procedures. To confirm intervention enactment, at the beginning of each intervention visit, study staff ask the participant what questions they have on how to complete the C-SCAT, and whether they want another practice session. These processes will ensure consistency of intervention delivery and integrity of data collection.

### Outcome Measures

#### Primary Outcome Measures

Self-efficacy is measured with the Patient-Reported Outcomes Measurement Information System Self-efficacy of Symptom Management Scale (PROMIS S-E), a domain-based 28-item scale. Items assess the person’s current level of confidence to manage or control symptoms, manage symptoms in different settings, and keep symptoms from interfering with work, sleep, relationships, or recreational activities [44]. Response options are on a 5-point Likert scale (1=*I am not confident at all* and 5=*I am very confident*). Scores are summed across items and can range from 28 to 140, and higher scores indicate greater self-efficacy. Cronbach α in our previous study with AYAs was 0.94 [45].

Symptom self-management is measured with the Symptom Self-management Behaviors Tool (SSMBT) [46]. The SSMBT contains 12 items with two subscales: manage symptoms (8 items) that address behaviors related to managing their symptoms and talk to health care provider (4 items) that address behaviors related to communicating with their HCP about their symptoms. Response options are on a 5-point Likert scale (0=*never* and 4=*always*). Scores are summed across all items. Higher scores indicate higher engagement in symptom self-management behaviors. The SSMBT was developed for AYAs with cancer and has evidence of being reliable and valid [46]. Reliability for the total scale was *α*= of .74. Construct validity was supported via principal component factor analysis and discriminant validity.

#### Secondary Outcome Measures

Symptom severity and distress in the past week are assessed using the MSAS [42]. Participants rate the severity on a 4-point Likert scale (1=*slightly* and 4=*very severe*) and the distress on a 5-point Likert scale (0=*none* and 4=*very much*). The adult version of the MSAS was used because it has 2 more symptoms (feeling bloated and problems with sexual interest or activity) than the MSAS 10‐18. In our previous study, AYAs indicated that these symptoms were relevant to them.

Two aspects of social function are assessed: quality of life related to social function and satisfaction with social function. Quality of life related to social function is assessed with the PROMIS SF (version 2.0) Ability to Participate in Social Roles and Activities. It contains 8 items, is scored on a 5-point Likert response scale (5=*never* and 1=*always*), and has strong evidence of reliability and validity in clinical populations, including individuals with cancer [47]. Satisfaction with social function is assessed with the PROMIS SF version 2.0 Satisfaction with Social Roles and Activities. It contains 8 items, is scored on a 5-point Likert response scale (1=*not at all* and 5=*very much*), has been validated in adults aged ≥18 years [48], and has been used in young adults with cerebral palsy [49].

#### Descriptive Measures

Self-reported demographic data are collected with the Biomedical Research Informatics Computing System, National Institute of Nursing Research [50] demographic form, including education, primary caregiver, employment, marital status, and household member count. Additional items assess household income, number of children, people living with the AYA, and living arrangement changes during treatment. Self-reported race and ethnicity are also being collected. Clinical and other descriptive variables are collected by study staff from the participant’s electronic health record and include age, sex, cancer diagnosis, time since diagnosis, chemotherapy protocol start date, treatment protocol, comorbid conditions, insurance type, and ZIP code.

#### Intervention Acceptability

Participants in the intervention group are asked to complete the C-SCAT Intervention Acceptability Survey. This 7-item survey assesses the acceptability of various aspects of the intervention, such as “How much effort did it take to do the CSCAT? and “How confident did you feel that you could complete the C-SCAT as instructed?”

### Data Collection

Data collection occurs at 3 time points (times 0, 1, and 2) at scheduled inpatient or outpatient visits (Figure 2). Baseline measures are collected at time 0, postintervention measures at time 1, and follow-up measures at time 2. Participants complete measures on a study-dedicated tablet computer using REDCap, or via a link to REDCap that is emailed to them. Study staff obtain clinical data from the electronic health record. The time 1 visit for the C-SCAT group is scheduled 2 weeks±7 days (to accommodate schedule changes) after completion of the intervention to allow time for the participant to have been at home (or outside the clinical setting) to self-manage their symptoms following chemotherapy. The time 1 visit for the UCCG group is scheduled 2 weeks±7 days (to accommodate schedule changes) after completion of the attention control visits. For both groups, the time 1 visit occurs during a scheduled inpatient or outpatient visit. Participants who are not scheduled for an inpatient or outpatient visit during the ±7-day window are contacted via telephone to complete the study measures and are sent a REDCap link via email. For both groups, the time 2 visit is scheduled 4 weeks±7 days from Time 1, and the same procedures for data collection as described earlier for time 1 are used.

### Safety

#### Data Safety Monitoring

This study carries less than minimal risk to participants. As this study is a multisite clinical trial and involves adolescents aged ≥15 years, we use a Safety Monitoring Committee to assess the safety of the study and monitor the data. The Safety Monitoring Committee consists of 2 members who have areas of expertise in AYAs with cancer and biostatistics. The Safety Monitoring Committee was established through the primary study site.

#### Monitoring Participant Safety

The research team uses a clearly defined protocol to monitor participant safety. Participant safety is monitored monthly and with each occurrence of a potential safety issue. The principal investigator, in collaboration with coinvestigators or site principal investigators, is responsible for monitoring the safety of participants at each site.

### Data Analysis Plan

#### Overview

This study is a 2-group (C-SCAT vs control), longitudinal randomized multicenter (5 sites), clinical trial with data collected at 3 time points (times 0, 1, and 2). Two primary variables are being evaluated for group differences in aim 1: symptom self-efficacy as measured by the PROMIS Self-Efficacy of Symptom Management Scale (PROMIS S-E) and self-management behaviors as measured by the SSMBT. The secondary outcomes in aim 2 are symptom severity, symptom distress, social function, and satisfaction with social function. The plan is to enroll 126 AYAs with cancer in active treatment from 5 sites. The study biostatistician who was not involved with data collection and will be blinded to group affiliation will conduct all analyses.

#### Sample Size and Power

A mixed effects linear model is planned to test for group differences across time for aims 1 and 2. The co-primary outcome variables from aim 1 are the PROMIS S-E and the SSMBT. From the outset, we planned to take a more conservative approach to the power analysis due in part to the limited number of SSMBT samples observed from only one site. Thus, for both primary variables, power was estimated using a 20% inflated variance estimate and a target power of 90% (as opposed to the usual 10% inflated variance and 80% power). A 20% inflated estimate of the residual SE was estimated as 12.73 for PROMIS S-E and 6.35 for SSMBT. Assuming the smallest clinically significant difference between the groups of 10 for PROMIS S-E and 4.8 for SSMBT, a Bonferroni adjusted alpha of .05/2=.025 and a power of 90%, PASS (Power Analysis & Sample Size, PASS 2022; NCSS, LLC [51]) software estimated the total sample size to be 77 for PROMIS S-E and 83 for SSMBT. However, the proposed model will have effects for time (0, 1, and 2), group (C-SCAT vs usual care), time-by-group interaction, site (1, 2, 3, 4, and 5), site-by-group interaction, and population (adolescent vs young adult). Accounting for the 16 degrees of freedom necessary to fit these model effects, the total sample size will be 83+16=99 who complete time 2. Allowing for an attrition as high as 20%, the final sample size will be 126 AYAs or approximately 25 per site. Recruitment in our preliminary multisite studies demonstrated that this sample size is feasible within the proposed period of the project.

Descriptive statistics will be computed for demographic and all outcome variables. All data will be examined graphically to determine the distributional properties. In cases of nonnormally distributed data, transformations may be used for analyses.

#### Aims

Aim 1 is to determine the effects of the C-SCAT versus usual care on the primary outcomes of self-efficacy for symptom management and symptom self-management behaviors immediately postintervention (time 1) and at follow-up 1 month later (time 2). Self-efficacy for symptom management and symptom self-management behaviors will be assessed at times 0, 1, and 2 and will be compared between the C-SCAT intervention group and the UCCG. We anticipate using a linear mixed effects model to test the effect of the C-SCAT versus usual care. The general form of the mixed linear model [52-54] is given by y=XB+ZU+ e, where y is the observed data vector (PROMIS S-E and SSMBT), X is a known design matrix, B is a vector of unknown fixed effects, Z is a known design matrix, U is a vector of unknown random effects, and e is a vector of unknown random errors. This model will include a random effect for participant (represented by ZU in the equation) and fixed effects for group (C-SCAT and UCCG), time (0, 1, and 2), group-by-time interaction, site (1, 2, 3, 4, and 5), group-by-site interaction, and population (adolescent and young adult; represented by XB in the equation). The model is flexible enough to accommodate the potential cofactors of race (eg, Black and White), ethnicity (eg, Hispanic and non-Hispanic), and gender (eg, male and female). The first step in testing aim 1 will be to test each of the primary variables (PROMIS S-E and SSMBT) using a contrast on the group-by-time interaction to specifically test for an immediate effect of C-SCAT versus the UCCG at time 1. A Bonferroni correction (*α*=.05/2=.025) is planned to adjust for testing each of the 2 primary variables. All other tests, for example, testing for a sustained effect at time 2, are considered supportive and will be testing with an *α* of .05. Modeling assumptions such as normality and homoscedasticity will be checked for each model. In the case of positively skewed data, a transformation (such as a log transformation) will be used to normalize the data. It is anticipated that all analyses will be performed using JMP (JMP 18, 2025 JMP 18; JMP Statistical Discovery LLC [55]) and SAS/STAT (Statistical Analysis Software Institute [56]) software, specifically PROC MIXED.

Aim 2 is to examine the effects of the C-SCAT versus usual care on secondary outcomes (symptom severity, symptom distress, quality of life related to social function, and satisfaction with social function) immediately postintervention (time 1) and at follow-up 1 month later (time 2). The mixed effects linear model presented in the aim 1 analysis plan is anticipated to be sufficient for the analysis of the secondary outcomes in aim 2. The secondary outcomes are considered supportive of the tests of an immediate effect (aim 1) and will be tested with an *α*= of .05.

#### Missing Data

In addition to strategies to prevent missing data, such as completion checks after study visits and reminders, we will monitor for patterns of missing data when data are merged on a quarterly schedule and determine reasons for missing data. We will use mean imputation for any missing data within a scale. Following an intention-to-treat principle, participants with any postintervention data will be included in the analysis.

#### Data Sharing Plan

Data will be shared with the scientific community via publishing results from the study in relevant peer-reviewed scientific journals and meetings. Analytic approaches will be available upon request.

## Results

Recruitment began at the first site on January 4, 2024. All sites were open to recruitment by May 1, 2024. For the quarterly benchmark goal for recruitment, enrollment is at approximately 73%. We anticipate that the study will close to recruitment in Fall 2026, and the final participant will complete the study by Spring 2027. We anticipate publishing results in 2027.

## Discussion

### Anticipated Findings

We describe the protocol of a RCT that is testing the effectiveness of the C-SCAT intervention designed to improve symptom self-management by helping AYAs understand their unique symptom experience and discuss it with their HCPs. The intervention aims to increase self-efficacy for managing symptoms and engagement in effective symptom self-management behaviors. Empowering patients to adopt a more active role in self-management during cancer treatment may lessen their symptom severity and distress.

### Limitations

The main limitations of this study center around reaching recruitment goals. Recruitment and retention are well-recognized challenges in any RCT with multiple data collection time points. However, beyond that, the nature of this study population poses additional challenges. Cancer in AYAs is fortunately considered rare, but this limits the number of eligible patients available. Recruiting minors (individuals aged <18 y) adds an extra layer of complexity to the consent process as both a parent or legally authorized representative and the eligible minor patient must be interested in study participation, the parent or legally authorized representative must give permission, and the patient must give assent. Sometimes the parent or legally authorized representative is interested, but the patient feels too ill or feels that the burden of participation is excessive. Sometimes the patient is interested, but the parent or legally authorized representative feels that the burden of participation may be excessive and wishes to protect the patient from that. Some potential participants may decline study participation because they do not perceive potential personal benefit even if randomized to the intervention arm, as they feel that their symptom experience is being well-managed and communication with their providers is already good. Alternatively, some potential participants may decline study participation because they feel too unwell from their symptom experience, or most commonly, express a sense of being overwhelmed, of ‘having a lot going on’ living a life that now includes cancer and cancer treatment. We aim to minimize recruitment challenges through our team’s expertise and experience with conducting several previous studies with this AYA population. In addition, we are recruiting from major cancer centers that have large catchment areas. To aid retention, both groups (C-SCAT and UCCG) participants receive personal contact with a study team member at least three times during the intervention period.

### Conclusions

In conclusion, because undermanaged symptoms reduce quality of life and increase symptom severity and distress, effective person-centered symptom self-management is imperative for AYAs with cancer. A major barrier, however, is a lack of effective symptom self-management interventions that are tailored to this population. The proposed study addresses this gap by testing an intervention designed to improve symptom self-management behaviors in AYAs with cancer who are in active treatment. If shown to be effective, the C-SCAT could be incorporated into clinical settings at the point of care and potentially into the electronic health record as an efficient, low-cost approach to improve symptom self-management.

## Abbreviations

AYA: adolescent and young adult
C-SCAT: Computerized Symptom Capture Tool
HCP: health care provider
HIPAA: Health Insurance Portability and Accountability Act
IFSMT: Individual and Family Self-Management Theory
RCT: randomized controlled trial
REDCap: Research Electronic Data Capture
S-E: PROMIS Self-efficacy of Symptom Management Scale
SSMBT: Symptom Self-management Behavior Tool
UCCG: usual care control group
